# Changes in Documentation Due to Patient Access to Electronic Health Records: Protocol for a Scoping Review

**DOI:** 10.2196/46722

**Published:** 2023-08-28

**Authors:** Eva Meier-Diedrich, Gail Davidge, Maria Hägglund, Anna Kharko, Camilla Lyckblad, Brian McMillan, Charlotte Blease, Julian Schwarz

**Affiliations:** 1 Brandenburg Medical School Immanuel Hospital Rüdersdorf University Clinic for Psychiatry and Psychotherapy Rüdersdorf Germany; 2 Faculty for Health Sciences Brandenburg Medical School Neuruppin Germany; 3 Centre for Primary Care and Health Services Research University of Manchester Manchester United Kingdom; 4 Department of Women’s and Children’s Health Uppsala University Uppsala Sweden; 5 Faculty of Health University of Plymouth Plymouth United Kingdom; 6 Department of Archives, Libraries, and Museums Uppsala University Uppsala Sweden; 7 Division of General Medicine Beth Israel Deaconess Medical Center Harvard Medical School Boston, MA United States

**Keywords:** electronic health record, patient-accessible electronic health record, patient portal, electronic portal, scoping review, patient access, documentation change, natural language processing, patient-clinician relationship, online record access, review method, library science, librarian

## Abstract

**Background:**

Internationally, patient-accessible electronic health records (PAEHRs) are increasingly being implemented. Despite reported benefits to patients, the innovation has prompted concerns among health care professionals (HCPs), including the possibility that access incurs a “dumbing down” of clinical records. Currently, no review has investigated empirical evidence of whether and how documentation changes after introducing PAEHRs.

**Objective:**

This paper presents the protocol for a scoping review examining potential subjective and objective changes in HCPs documentation after using PAEHRs.

**Methods:**

This scoping review will be carried out based on the framework of Arksey and O’Malley. Several databases will be used to conduct a literature search (APA PsycInfo, CINAHL, PubMed, and Web of Science Core Collection). Authors will participate in screening identified papers to explore the research questions: How do PAEHRs affect HCPs’ documentation practices? and What subjective and objective changes to the clinical notes arise after patient access? Only studies that relate to actual use experiences, and not merely prior expectations about PAEHRs, will be selected in the review. Data abstraction will include but will not be limited to publication type, publication year, country, sample characteristics, setting, study aim, research question, and conclusions. The Mixed Methods Appraisal Tool will be used to assess the quality of the studies included.

**Results:**

The results from this scoping review will be presented as a narrative synthesis structured along the key themes of the corpus of evidence. Additional data will be prepared in charts or tabular format. We anticipate the results to be presented in a scoping review at a later date. They will be disseminated at scientific conferences and through publication in a peer-reviewed journal.

**Conclusions:**

This is the first scoping review that considers potential change in documentation after implementation of PAEHRs. The results can potentially help affirm or refute prior opinions and expectations among various stakeholders about the use of PAEHRs and thereby help to address uncertainties. Results may help to provide guidance to clinicians in writing notes and thus have immediate practical relevance to care. In addition, the review will help to identify any substantive research gaps in this field of research. In the longer term, our findings may contribute to the development of shared documentation guidelines, which in turn are central to improving patient communication and safety.

**International Registered Report Identifier (IRRID):**

PRR1-10.2196/46722

## Introduction

### Background

Electronic health records (EHRs) are common in almost all areas of health care and are an indispensable tool for saving and sharing information between health care professionals (HCPs) [1]. A more recent development focuses on opening clinical notes or entire EHRs for patients [2] and their proxies [3-7]. These so-called patient-accessible electronic health records (PAEHRs) are well established internationally, especially in Scandinavian countries and the United States, but are not yet fully embedded even among high-income countries [8-11]. An essential part of patients’ online record access (ORA) through a PAEHR is access to the clinical free-text notes written by clinicians. Giving patients access to these notes is often referred to as “open notes” in the literature [2].

Research shows that HCPs are often skeptical about giving patients ORA [12,13], and many of their concerns relate to how PAEHR use might impact their clinical routines, workload, and patient safety [11,13-20]. Regarding documentation, many HCPs anticipate changing the content and tone of their notes when patients have ORA, which, it is feared, might ultimately compromise the integrity of their records [11,19,21]. For example, a tendency to avoid technical terminology to facilitate patient understanding could have a negative impact on multidisciplinary communication within the team [21-24]. Also, some HCPs feel that they may be less detailed or less candid in their documentation and need to omit information or even start using parallel documentation (a “shadow record”) to protect patients from information that they consider as potentially harmful or disruptive [14,15,20,25-27]. In contrast, however, there are other voices that assume the introduction of PAEHRs could make notes more patient-friendly by using a more patient-centered and less stigmatizing language and could also stimulate communication between HCP and patients [22].

As Blease et al [11] noted, while most studies explore subjective changes after introducing open notes, there are few studies demonstrating objective changes, and where these studies exist, they offer inconclusive results [22,28,29] and are often hampered by methodological limitations. There is a growing body of qualitative research [30] as well as research using natural language processing approaches to explore the language used by clinicians in their records, including the potential for stigmatizing language [31]. However, it is unclear from these studies whether access affects, or indeed, even improves the quality of recordkeeping with the knowledge that patients may read what the clinician has written [31]. Despite the increasing scientific interest and debate within medicine, little is currently known about how far sharing EHRs with patients affects clinical documentation [11,32].

### Study Objectives

The objective of the proposed scoping review is to identify, collate, and evaluate possible changes of documentation after implementing patient access to EHRs. The scoping review focuses exclusively on studies including postimplementation data, such as experiences of the stakeholders (HCPs, patients, policymakers and designers of EHRs or patient portals), while excluding expectations prior to implementation.

As outlined, HCPs are often reluctant, or even critical of giving patients ORA, and expect an additional documentation burden through their introduction. To address these obstacles to implementation, it is timely and appropriate to review the existing body of literature and summarize what is currently known regarding PAEHR documentation change. This scoping review is intended to increase knowledge for stakeholders about the kinds of documentation changes that might arise with PAEHRs, illuminate how this relates to documentation practices, provide recommendations for future clinical practice, and identify further research gaps.

## Methods

### Scoping Review

Compared with the systematic review method, which is guided by a strongly focused research question, a scoping review aims to open up the spectrum of the available evidence on a relatively new field of research, so that its breadth and depth become visible [33]. We will conduct a scoping review following the framework proposed by Arksey and O’Malley [33]. Their approach consists of the following five stages: (1) identifying the research question, (2) identifying the relevant studies, (3) selecting eligible studies, (4) collecting data, and (5) summarizing data and synthesizing results. The review will be reported following the PRISMA (Preferred Reporting Items for Systematic Reviews and Meta-Analyses) Extension for Scoping Reviews checklist [34,35]. Any subsequent modification of the study design will be highlighted in the final publication, which is aimed at being published in a JMIR journal.

### Stage 1: Identifying the Research Question

Through discussions with the research team, we decided on the following research questions: Does clinical documentation change after the introduction of ORA for patients? If so, what objective and subjective changes arise after PAEHR implementation? By objective, we mean such differences that can be demonstrated by a direct quantifiable comparison of clinical notes before and after implementation of PAEHRs. By subjective, we refer to clinicians’ perceptions of how they write their notes after PAEHR implementation. In the context of this scoping review, we define PAEHR to be any channel in which patients have electronic access to their patient record (eg, through the internet or via patient portals and apps).

### Stage 2: Identifying Relevant Studies

**Figure 1:**
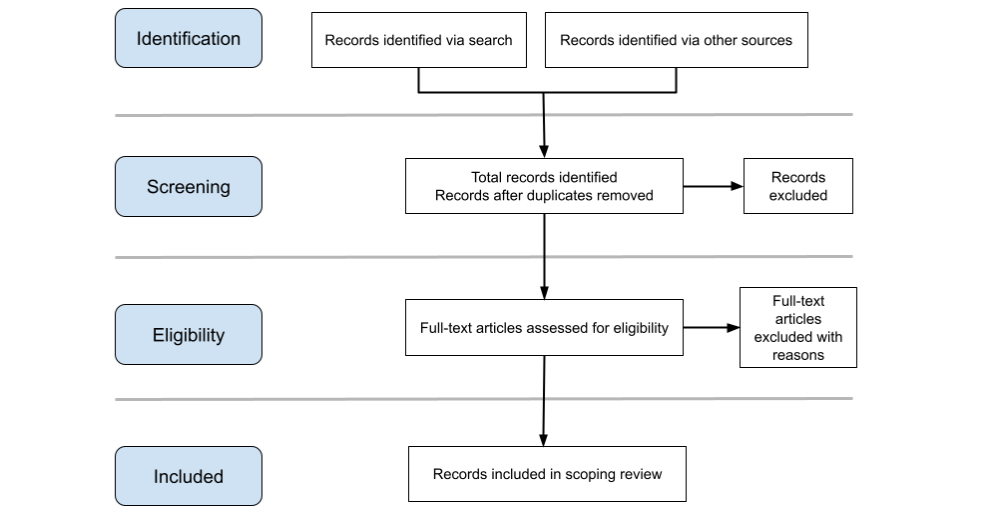
Flowchart describing the multistage screening process.

The process of identifying relevant studies is outlined in the flowchart (see Figure 1). The deduplication process will be carried out by an experienced research librarian at Uppsala University, Sweden. In advance, the research team will conduct a rigorous manual search to obtain a basic overview of the available evidence and to refine the scope of the review as well as the search strategy as Popay et al suggest [36].

The literature search in the following 4 databases will be conducted by the librarian Malin Barkelind from Uppsala University: APA PsycInfo, CINAHL, PubMed, and Web of Science Core Collection. The search strategy was developed in collaboration with the Uppsala University library and consists of three key concepts: (1) EHRs, (2) sharing EHRs with patients, and (3) changes in documentation, which were combined with the Boolean AND (Textbox 1). The search terms were adapted according to different databases. The complete search string is stored in Multimedia Appendix 1. In addition, we will include individual relevant records from the hand search conducted previously.

**Key concepts of the search strategy.**

**Electronic health record search string**

- “inpatient portal*” OR “open notes” OR opennotes OR PAEHR OR “patient portal*” OR “patient web portal*” OR “Electronic Health Records”
- “clinic notes” OR “clinical notes” OR “progress notes” OR “doctors notes” OR EHR OR “health record*” OR “health care record*” OR “medical record*” OR “mental health notes” OR “patient record*” OR “psychiatric notes” OR “psychotherapy notes” OR “visit notes”

**Sharing electronic health records with patients search string**

- “guardian access” OR “parental access” OR “parents access” OR “patient access*” OR “patients access*” OR “patient online access” OR “patients online access” OR “proxy access” OR “shared medical record*” OR “shared health record*”

**Documentation changes search string**

- “Language”[Mesh] OR “Attitude”[Mesh] OR “Comprehension”[Mesh]
- accura* OR ambigu* OR characteristics OR characters OR clarity OR content* OR completeness OR comprehend* OR comprehensibl* OR comprehension* OR correctness OR dialog* OR express* OR directness OR impression* OR inaccura* OR incomplete* OR incomprehen* OR incorrectness* OR intelligib* OR interpret* OR intuitive* OR language OR length OR linguistic* OR misconception* OR misinterpret* OR misread* OR misunderstand* OR monolog* OR negative* OR pattern* OR positive* OR pronoun* OR readab* OR style* OR simplicity OR terminolog* OR transparen* OR truthful* OR unambigu* OR understand* OR untruthful* OR veracity OR wordcount* OR words OR writing
- OR attitude* OR emotion* OR experience* OR perception* OR satisfact*
- OR adopt* OR alter* OR censor* OR change* OR changing OR difference* OR introduc* OR implement* OR modif* OR postimplement*

### Stage 3: Selecting Eligible Studies

#### Inclusion and Exclusion Criteria

Inclusion and exclusion criteria (Textbox 2) were defined by the entire research team and will be applied in the study selection process. Due to the limited number of publications available on the subject, there will be no restrictions on the study type. As PAEHRs are only gradually being implemented in various countries, we will refrain from any location restrictions. A wide variety of approaches exist to make clinical notes available to patients electronically [37]. We will include all studies examining the actual implementation and the use of patient ORA regardless of the digital device used (eg, web-based and mobile apps). Varieties of studies exploring the sharing of hard copies of patients’ clinical records will be excluded.

**Inclusion and exclusion criteria.**

**Inclusion criteria**

- Study design: all study types
- Publication: original, peer-reviewed work including empirical data published between January 1, 2005, and June 30, 2023 in English
- Study location: all medical disciplines, all health care settings, no location restrictions
- Study participants: patients and health care professionals of all ages
- Studies that examine actual use by stakeholders and their experiences with patient-accessible electronic health records

**Exclusion criteria**

- Paper-based, disc, or USB sharing of patients’ records
- Papers without empirical data (eg, comments, editorials, news)
- Gray data (websites, tweets, blogs)
- Studies that exclusively investigate expectations about patient-accessible electronic health records

#### Study Selection Process

We will use Rayyan Software (Rayyan Systems, Inc) for conducting a collaborative, blinded title and abstract screening [38]. All members of the research team will participate in this process and each record of the result set will be evaluated by at least 2 people. Discrepancies will be discussed, taking the full texts of the corresponding studies into account. In case of disagreements that cannot be resolved, a third reviewer will be involved and entrusted with the decision of including or excluding the study.

### Stage 4: Collecting Data

After selecting the studies to include, metadata (eg, title, authors, and publication year) of the remaining records will be exported and summarized in a Google Sheets (Google LLC) spreadsheet for further processing. To extract and organize relevant data from included studies, the spreadsheet will be extended by the following and other parameters based on the studies’ full text: country, study design, sample, characteristics of study participants (eg, gender, age, ethnicity, type of stakeholder), treatment setting and medical specialty, and study purpose. Data extraction will be performed involving all members of the research team. Furthermore, the first author will check the data extraction for correctness and completeness. To assess the quality and methodological rigor of the studies, the Mixed Methods Appraisal Tool (MMAT) will be used [39]. Two researchers will independently conduct the MMAT grading of all studies and consent their results. If no agreement can be reached, a third independent researcher will be consulted.

### Stage 5: Summarizing Data and Synthesizing Results

#### Narrative Synthesis

Study results will be extracted from the full texts by the lead author and summarized in (1) a reduced format within a textbox, providing an overview of the findings from all included studies, and (2) a detailed version for narrative synthesis. The latter will be analyzed independently by at least 2 researchers using thematic analysis [40]. Objective and subjective changes of HCPs’ documentation practices after the introduction of patient ORA will be used as guiding deductive themes and are informed by the research question but may change in the analytical process. As Levac et al [35] suggest, we aim to identify patterns and relationships within and across studies to identify potential factors influencing documentation after PAEHR implementation. In assessing the methodological rigor of the studies, we also envisage the potential to identify research gaps; for example, we predict there may be a preponderance of survey research investigating clinicians’ perceptions about documentation changes rather than studies investigating objective markers of any such documentation changes. While the former studies may be useful, they may be compromised by responder biases. Results will then be discussed and approved by the entire research team.

#### Assessing the Robustness of the Synthesis

As Popay et al [36] state, the robustness of the narrative synthesis depends on the quality of included studies as well as on the trustworthiness of the synthesis. In order to minimize bias, we will conduct the study quality appraisal through MMAT to ensure that studies of equal technical quality are given equal weight. To provide a high level of trustworthiness in our review, reviewers will have detailed information about the eligibility criteria and the type of intervention (PAEHR) in order to provide sufficient information for replication.

### Ethical Considerations

Since we will use only publicly available data material with the scoping review methodology, this study is not subject to ethical approval.

## Results

The main results from our analysis will be presented in a narrative form, focusing on subjective and objective changes in clinical notes as well as on changes in HCPs’ documentation after sharing EHRs with patients. Additional data on year, country, study design, characteristics of study participants, setting, sample, medical specialty, and study purpose will be presented in diagrams or tabular format.

## Discussion

Research indicates that HCPs have concerns regarding the effects of ORA on clinical routines, workload, and patient safety as patients increasingly gain access to medical information on the internet [11-20]. Although a limited number of studies have examined alterations in documentation practices following the implementation of policies, these studies have primarily focused on subjective evaluations rather than objective assessments [11]. Furthermore, the few studies attempting to investigate objective changes in documentation have yielded inconclusive results that are further restricted by methodological limitations [22,28,29].

Although there are already a few reviews that address the use of patient ORA [32,41-43], no review specifically addresses the potential changes to clinical documentation that may result from the implementation and use of PAEHR. Our scoping review aims to map current research into documentation changes and to potentially raise awareness among many different involved parties about the risks and opportunities of PAEHR use.

A potential limitation of the study is a reduced depth of the analysis due to the broader nature of the scoping review. In addition, due to exclusion of gray literature, it is possible that some studies will be overlooked. Nevertheless, we defined our search strategy to identify the most comprehensive and high-quality evidence.

Dependent on the findings, this study may offer important insights on how to support effective documentation practice in the future. For example, the findings may provide a basis for a widely demanded “documentation training” that could better prepare HCPs and patients on how to read and write notes [20]. The scoping review will strive to identify any existing research gaps and to indicate directions for further studies in this field. The growing body of evidence on natural language processing in relation to documentation changes after PAEHR introduction will be explored.

## Appendix

Multimedia Appendix 1 Adapted search strategies for each database.

## Abbreviations

EHR: electronic health record
HCP: health care professional
MMAT: Mixed Methods Appraisal Tool
ORA: online record access
PAEHR: patient-accessible electronic health record
PRISMA: Preferred Reporting Items for Systematic Reviews and Meta-Analyses

## Data Availability

The data used for the scoping review are openly available and can be retrieved from the following databases: APA PsycInfo, CINAHL, PubMed, and Web of Science Core Collection.
