# Supplementary material for: Changes in Documentation Due to Patient Access to Electronic Health Records: Protocol for a Scoping Review
Source: JMIR Res Protoc. 2023 Aug 28;12:e46722. doi: 10.2196/46722 (PMC10495856; doi:10.2196/46722)
Supplement: Multimedia Appendix 1 [file resprot_v12i1e46722_app1.docx]

## PubMed

| **Search no.** | **Search terms** |
| --- | --- |
| 1 | "Patient Portals"[MeSH] |
| 2 | "inpatient portal*" OR "open notes" OR opennotes OR PAEHR OR "patient portal*" OR "patient web portal*" [Title/Abstract] |
| 3 | #1 OR #2 |
| 4 | "Electronic Health Records"[MeSH] |
| 5 | "clinic notes" OR "clinical notes" OR "progress notes" OR "doctors notes" OR EHR OR "health record*" OR "healthcare record*" OR "medical record*" OR "mental health notes" OR "patient record*" OR "psychiatric notes" OR "psychotherapy notes" OR "visit notes" [Title/Abstract] |
| 6 | #4 OR #5 |
| 7 | "Patient Access to Records"[Mesh] |
| 8 | "guardian access" OR "parental access" OR "parents access" OR "patient access*" OR "patients access*" OR "patient online access" OR "patients online access" OR "proxy access" OR "shared medical record*" OR "shared health record*" [Title/Abstract] |
| 9 | #7 OR #8 |
| 10 | #6 AND #9 |
| 11 | #3 OR #10 |
| 12 | "Language"[Mesh] OR "Attitude"[Mesh] OR "Comprehension"[Mesh] |
| 13 | accura* OR adopt* OR alter* OR ambigu* OR attitude* OR censor* OR change* OR changing OR characteristic* OR characters OR clarity OR completeness OR comprehend* OR comprehensib* OR comprehension* OR content* OR correctness OR dialog* OR difference* OR directness OR emotion* OR experience* OR express* OR implement* OR impression* OR inaccura* OR incomplete* OR incomprehen* OR incorrectness* OR intelligib* OR interpret* OR introduc* OR intuitive* OR language OR length OR linguistic* OR misconception* OR misinterpret* OR misread* OR misunderstand* OR modif* OR monolog* OR negative* OR pattern* OR perception* OR positive* OR postimplement* OR pronoun* OR readab* OR satisfact* OR simplicity OR style* OR terminolog* OR transparen* OR truthful* OR unambigu* OR understand* OR untruthful* OR veracity OR wordcount* OR words OR writing [Title/Abstract] |
| 14 | #12 OR #13 |
| 15 | #11 AND #14 |
| 16 | #15 AND "english"[Language] |
| 17 | #16 NOT ("address"[Publication Type] OR "comment"[Publication Type] OR "editorial"[Publication Type] OR "news"[Publication Type]) |

## Web of Science

| **Search no.** | **Search terms** |
| --- | --- |
| 1 | TS=("inpatient portal*" OR "open notes" OR opennotes OR PAEHR OR "patient portal*" OR "patient web portal*") |
| 2 | TS=("clinic notes" OR "clinical notes" OR "progress notes" OR "doctors notes" OR EHR OR "health record*" OR "healthcare record*" OR "medical record*" OR "mental health notes" OR "patient record*" OR "psychiatric notes" OR "psychotherapy notes" OR "visit notes") |
| 3 | TS=("guardian access" OR "parental access" OR "parents access" OR "patient access*" OR "patients access*" OR "patient online access" OR "patients online access" OR "proxy access" OR "shared medical record*" OR "shared health record*") |
| 4 | #2 AND #3 |
| 5 | #1 OR #4 |
| 6 | TS=(accura* OR adopt* OR alter* OR ambigu* OR attitude* OR censor* OR change* OR changing OR characteristic* OR characters OR clarity OR completeness OR comprehend* OR comprehensib* OR comprehension* OR content* OR correctness OR dialog* OR difference* OR directness OR emotion* OR experience* OR express* OR implement* OR impression* OR inaccura* OR incomplete* OR incomprehen* OR incorrectness* OR intelligib* OR interpret* OR introduc* OR intuitive* OR language OR length OR linguistic* OR misconception* OR misinterpret* OR misread* OR misunderstand* OR modif* OR monolog* OR negative* OR pattern* OR perception* OR positive* OR postimplement* OR pronoun* OR readab* OR satisfact* OR simplicity OR style* OR terminolog* OR transparen* OR truthful* OR unambigu* OR understand* OR untruthful* OR veracity OR wordcount* OR words OR writing) |
| 7 | #5 AND #6 |
| 8 | #7 AND (LA==("ENGLISH")) |
| 9 | #8 NOT (DT==("EDITORIAL MATERIAL")) |

## PsycInfo

| **Search no.** | **Search terms** |
| --- | --- |
| 1 | TI OR AB "inpatient portal*" OR "open notes" OR opennotes OR PAEHR OR "patient portal*" OR "patient web portal*" |
| 2 | DE "Electronic Health Records" |
| 3 | TI OR AB "clinic notes" OR "clinical notes" OR "progress notes" OR "doctors notes" OR EHR OR "health record*" OR "healthcare record*" OR "medical record*" OR "mental health notes" OR "patient record*" OR "psychiatric notes" OR "psychotherapy notes" OR "visit notes" |
| 4 | #2 OR #3 |
| 5 | TI OR AB "guardian access" OR "parental access" OR "parents access" OR "patient access*" OR "patients access*" OR "patient online access" OR "patients online access" OR "proxy access" OR "shared medical record*" OR "shared health record*" |
| 6 | #4 AND #5 |
| 7 | #1 OR #6 |
| 8 | DE "Adolescent Attitudes" OR DE "Adult Attitudes" OR DE "Attitudes" OR DE "Child Attitudes" OR DE "Client Attitudes" OR DE "Comprehension" OR DE "Counselor Attitudes" OR DE "Language" OR DE "Linguistics" OR DE "Parental Attitudes" OR DE "Phrases" OR DE "Pronouns" OR DE "Psychologist Attitudes" OR DE "Readability" OR DE "Sentences" OR DE "Terminology" OR DE "Vocabulary" OR DE "Written Language" |
| 9 | TI OR AB accura* OR adopt* OR alter* OR ambigu* OR attitude* OR censor* OR change* OR changing OR characteristic* OR characters OR clarity OR completeness OR comprehend* OR comprehensib* OR comprehension* OR content* OR correctness OR dialog* OR difference* OR directness OR emotion* OR experience* OR express* OR implement* OR impression* OR inaccura* OR incomplete* OR incomprehen* OR incorrectness* OR intelligib* OR interpret* OR introduc* OR intuitive* OR language OR length OR linguistic* OR misconception* OR misinterpret* OR misread* OR misunderstand* OR modif* OR monolog* OR negative* OR pattern* OR perception* OR positive* OR postimplement* OR pronoun* OR readab* OR satisfact* OR simplicity OR style* OR terminolog* OR transparen* OR truthful* OR unambigu* OR understand* OR untruthful* OR veracity OR wordcount* OR words OR writing |
| 10 | #8 OR #9 |
| 11 | #7 AND #10 |
| 12 | #11 AND *Language: English* |
| 13 | #12 NOT ((ZZ "column/opinion") OR (ZZ "comment/reply") OR (ZZ "editorial")) |

## CINAHL

| **Search no.** | **Search terms** |
| --- | --- |
| 1 | (MH "Patient Portals") |
| 2 | "inpatient portal*" OR "open notes" OR opennotes OR PAEHR OR "patient portal*" OR "patient web portal*" [Title/Abstract] |
| 3 | #1 OR #2 |
| 4 | (MH "Electronic Health Records") |
| 5 | "clinic notes" OR "clinical notes" OR "progress notes" OR "doctors notes" OR EHR OR "health record*" OR "healthcare record*" OR "medical record*" OR "mental health notes" OR "patient record*" OR "psychiatric notes" OR "psychotherapy notes" OR "visit notes" [Title/Abstract] |
| 6 | #4 OR #5 |
| 7 | (MH "Patient Access to Records") |
| 8 | "guardian access" OR "parental access" OR "parents access" OR "patient access*" OR "patients access*" OR "patient online access" OR "patients online access" OR "proxy access" OR "shared medical record*" OR "shared health record*" [Title/Abstract] |
| 9 | #7 OR #8 |
| 10 | #6 AND #9 |
| 11 | #3 OR #10 |
| 12 | (MH "Language+") OR (MH "Attitude+") |
| 13 | accura* OR adopt* OR alter* OR ambigu* OR attitude* OR censor* OR change* OR changing OR characteristic* OR characters OR clarity OR completeness OR comprehend* OR comprehensib* OR comprehension* OR content* OR correctness OR dialog* OR difference* OR directness OR emotion* OR experience* OR express* OR implement* OR impression* OR inaccura* OR incomplete* OR incomprehen* OR incorrectness* OR intelligib* OR interpret* OR introduc* OR intuitive* OR language OR length OR linguistic* OR misconception* OR misinterpret* OR misread* OR misunderstand* OR modif* OR monolog* OR negative* OR pattern* OR perception* OR positive* OR postimplement* OR pronoun* OR readab* OR satisfact* OR simplicity OR style* OR terminolog* OR transparen* OR truthful* OR unambigu* OR understand* OR untruthful* OR veracity OR wordcount* OR words OR writing [Title/Abstract] |
| 14 | #12 OR #13 |
| 15 | #11 AND #14 |
| 16 | #15 *Narrow by Language: English* |
| 17 | #16 NOT ((ZT "commentary") OR (ZT "editorial") OR (ZT "newspaper") OR (ZT "opinion")) |
